# Serum amyloid A3 deficiency impairs in vitro and in vivo adipocyte differentiation

**DOI:** 10.1080/21623945.2021.1916220

**Published:** 2021-04-25

**Authors:** Ellen Vercalsteren, Christine Vranckx, Ines Vermeire, Max Gooijen, Roger Lijnen, Ilse Scroyen

**Affiliations:** a Center for Molecular and Vascular Biology, Department of Cardiovascular Sciences, KU Leuven, Leuven, Belgium; b Health Department, University Colleges Leuven Limburg, Leuven, Belgium

**Keywords:** Serum amyloid A3, adipogenesis, obesity, adipocyte differentiation, in vitro model, murine adipogenesis model

## Abstract

Obesity, caused by an excess adipose tissue, is one of the biggest health-threats of the 21^st^ century. Adipose tissue expansion occurs through two processes: (i) hypertrophy, and (ii) hyperplasia, the formation of new adipocytes, also termed adipogenesis. Recently, serum amyloid A3 (Saa3) has been implicated in adipogenesis. Therefore, the aim of this study was to investigate the effect of Saa3 on adipogenesis using both an in vitro and in vivo murine model. Saa3 gene silenced pre-adipocytes ha a lower expression of pro-adipogenic markers and less lipid accumulation, indicating impaired adipogenesis. Furthermore, male NUDE mice, injected with Saa3 gene silenced pre-adipocytes developed smaller fat pads with smaller adipocytes and lower expression of pro-adipogenic markers than their control counterparts. This confirms that Saa3 gene silencing indeed impairs adipogenesis, both in vitro and in vivo. These results indicate a clear role for Saa3 in adipogenesis and open new perspectives in the battle against obesity.

## Introduction

With a prevalence that will only keep on increasing and the high association with the development of co-morbidities, it is evident that obesity is one of the biggest health-challenges of the 21^st^ century [1]. The obese state is induced by an adipose tissue expansion. This expansion of adipose tissue occurs via two routes: hypertrophy, the enlargement of existing adipocytes, and hyperplasia, the formation of new adipocytes [2]. Adipocyte hypertrophy induces inflammation, because the hypertrophic adipocytes have become too large and can no longer function properly [3,4]. This adipose tissue inflammation affects the entire body because adipose tissue is such an important endocrine organ [5,6]. Ultimately, this leads to the development of co-morbidities, that can be life threatening.

One could argue that adipocyte hyperplasia is a rescue mechanism to decrease adipose tissue inflammation and dysfunction, since the newly formed adipocytes can take up excess lipids thereby relieving the stress exerted on the hypertrophic adipocytes [7,8]. However, this is a vicious cycle: these newly formed adipocytes also will become hypertrophic and thus aggravate the inflammatory state of the adipose tissue even further. To break this vicious cycle and thus curb adipose tissue dysfunction and its detrimental consequences it is of vital importance to understand this process of hyperplasia.

Serum amyloid A3 (Saa3) is abundantly expressed in obese adipose tissue and its role in adipose tissue inflammation has been quite well documented [9–11]. However, studies on the effect of Saa3 in adipogenesis have been contradictory. On the one hand, Saa3 has been implicated as a growth factor for adipocytes and inhibitor of adipocyte differentiation [12]. On the other hand, Zhao et al demonstrated that Saa3 induces Pparγ expression, which is known to be a key driver of adipogenesis [13]. Furthermore, dexamethasone and interleukin-6, pro-inflammatory molecules, induce Saa3 expression in adipocytes, indicating that Saa3 might be of particular importance for inflammation-induced adipogenesis [14]. Further elucidating the role of Saa3 in adipogenesis might aid in the search for new treatment strategies to decrease adipose tissue dysfunction. However, much more research is necessary to understand the role of Saa3 in adipogenesis. Therefore, the aim of this study was to unravel the role of Saa3 in adipogenesis using both in vitro and in vivo models.

## Materials and methods

### In vitro adipocyte differentiation model

#### Saa3 gene silencing

For all in vitro experiments, 3T3-F442A murine pre-adipocytes were used [15]. To create a pre-adipocyte cell line with a stable Saa3 gene silencing, the ‘MISSION shRNA lentiviral transduction particles’ system and the ExpressMag® transduction system (Sigma-Aldrich, St. Louis, MO, USA) were used as previously described [16]. To achieve Saa3 gene silencing, five different clones were tested (clone 1: GCTGCTAAAGTCATCAGTGAT; clone 2: TGGGTCCAGTTCATGAAAGAA; clone 3: GAGAGGCTGTTCAGAAGTTCA; clone 4: GCCTACTCTGACATGAAGAAA; clone 5: TGGGAGTTGACAGCCAAAGAT). MISSION non-target shRNA transduction particles (SHC002V) were used as negative control. Puromycin-resistant pre-adipocytes were differentiated into mature adipocytes as described below in the ‘differentiation’ section.

### Differentiation

3T3-F442A murine pre-adipocytes were grown in DMEM High-glucose medium (41965–062, Thermo Fisher Scientific, Inc., Waltham, MA, USA) supplemented with 10% bovine calf serum supplemented with iron (A3520501, Thermo Fischer Scientific Inc.) and 1% penicillin/streptomycin (10378016, Thermo Fisher Scientific Inc.) (=basal medium). At the start of the differentiation protocol, cells were seeded at 25 × 10^3^ cells/cm^2^ in 6-well plates. When cells reached confluence, their medium was switched to DMEM High-glucose medium supplemented with 10% foetal bovine serum (26140079, Thermo Fischer Scientific Inc.) and 1% penicillin/streptomycin (10378016, Thermo Fisher Scientific Inc.) (= basal differentiation medium). This time point was termed day 0. At day 2, cells were exposed to induction medium (Table 1). From day 4 until the end of the experiment, cells were cultured in differentiation medium (Table 1). During differentiation, RNA samples were collected at different time points and at the end of the differentiation, RNA was collected and an Oil Red O staining was performed as described previously [17]. Briefly, at the end of the differentiation protocol, cells were washed with phosphate-buffered saline (PBS) for 5 min and then fixed in 1.5% glutaraldehyde in PBS for 5 min. Hereafter, cells were stained with a 0.2% Oil Red O solution (Sigma-Aldrich) for 2 h at 37°C. After the staining, cells were washed and kept on tissue culture water until analysis. Images were taken at 50X and for spectrophotometric quantification, Oil Red O was extracted from the fixed cells using dimethylsulfoxide and absorbance was measured at 490 nm.

**Table 1.** Composition of the media used for in vitro adipocyte differentiation

Composition of media (final concentration)		
	Induction medium	Differentiation medium
Insulin	17 nM	17 nM
T3	2 nM	2 nM
Dexamethasone	100 µM	/
IBMX	100 µM	/

This table depicts the final concentration of the components added to the basal differentiation medium (DMEM High-glucose supplemented with 10% foetal bovine serum and 1% penicillin/streptomycin).

Abbreviation: IBMX, methylisobutylxanthine.

### In vivo adipocyte differentiation model

Athymic, male, 4-week-old, BALB/c Nude mice were purchased from Charles River Laboratories (Les Oncins, France). Mice were co-housed in micro-isolation cages (3–5 animals/cage) in a temperature-controlled environment (22°C) on a 12 h day/night cycle. After 1 week of acclimatization, mice were injected subcutaneously in the back with 10 × 10^6^ cultured 3T3-F442A pre-adipocytes transduced with either *Saa3* gene silencing particles (n = 7) or control particles (n = 7) in phosphate buffered saline (PBS) as described previously [18–20]. After 5 weeks of western diet (43% fat, 42% carbohydrate, 15% protein), mice were anesthetized with 60 mg/kg pentobarbital and de novo formed fat pads and GN and SC adipose tissue were weighed and stored at −80°C or fixed in 4% formaldehyde for subsequent analysis.

## Gene expression and histological analyses

### Gene expression assays

RNA extractions were performed using the RNeasy mini kit (Qiagen, Basel, Switserland) according to manufacturer’s instructions. 10 ng/µl RNA was used to transcribe into cDNA using the Multiscribe^TM^ Reverse Transcriptase kit (Thermo Fischer Scientific, Waltham, MA, USA) was used to transcribe according to the manufacturer’s protocol. Taqman gene expression assays (Thermo Fischer Scientific) or custom-designed primer-probe sets were used to analyse gene expression (Table 2) with quantitative RT-PCR according to a protocol previously described [21]. Data were obtained as cycle threshold (Ct) values and were analysed using the ∆∆Ct method.

**Table 2.** Overview of markers used for gene expression analysis

Markers detected via qPCR		
*Gene*	*Description*	*Assay number or primer-probe set*
Saa3	Serum Amyloid A3; chemoattractant properties	Mm00441203_m1
Pref1	Preadipocyte factor 1; marker for pre-adipocytes	Mm00494477_m1
C/EBPα	CCAAT/enhancer binding protein α; stimulates adipocgenesis	Mm00514283_s1
C/EBPβ	CCAAT/enhancer binding protein β stimulates adipocgenesis	Mm00843434_s1
C/EBPδ	CCAAT/enhancer binding protein δ stimulates adipocgenesis	Mm00786711_s1
Β-actin	Β-actin	Mm01205647_g1
Pparγ	Peroxisome proliferator-activated receptor gamma; regulator of adipogenesis and lipid storage	Sense (5’-3’): CTG TCG GTT TCA GAA GTG CCT
		Anti-sense (5’-3’): ATC TCC GCC AAC AGC TTC TC
		Probe (5’-3’): CCC AAA CCT GAT GGC ATT GTG AGA CA
Ap2	Adipocyte protein 2, aka FABP4, fatty acid binding protein 4; plays a role in fatty acid transport	Sense (5’-3’): CCT TCA AAC TGG GCG TGG
		Anti-sense (5’-3’): CGT TTT CTC TTT ATT GTG GTC GAC T
		Probe (5’-3’): ATG CTC TTC ACC TTC CTG TCG TCT GCG
GPDH	Glycerol-3-phosphate dehydrogenase; involved in lipid accumulation inside lipid droplets	Sense (5’-3’): GGT GGC AGA GGC CTT TG
		Anti-sense (5’-3’): TGC CCA TTT AGC ATC TCC TT
		Probe (5’-3’): TCG AAC TGG AAA GTC CAT TGA GCA GC

### Histological analysis

Adipocyte size was calculated on haematoxylin/eosin-stained (H&E) sections (7 µm) as follows: the area of interest was marked, excluding regions of stromal-vascular fractions that do not contain adipocytes. Subsequently, the individual adipocytes in this area of interest were manually counted. The average cell size was calculated as the area of interest divided by the adipocyte count in that area. The adipocyte density was calculated by dividing the amount of adipocytes by the area of interest and then multiplying this by 10^6^. For every fat pad, 10 images were analysed. Images were taken at 200x and analysed using the opensource image analysis software 2.0.0. rc-69/1.52p.

### Statistical analysis

Data are represented as mean ± standard error of the mean. Graphpad Prism 8 was used for statistical analysis (Graphpad Software Inc, San Diego, California, USA). Statistical significance was calculated using Mann-Whitney-U test to detect differences between two groups or two-way analysis of variance (ANOVA) to detect differences over time. P-values < 0.05 were considered statistically significant.

## Results

### Saa3 gene silencing impairs in vitro adipogenesis

**Figure 1.**
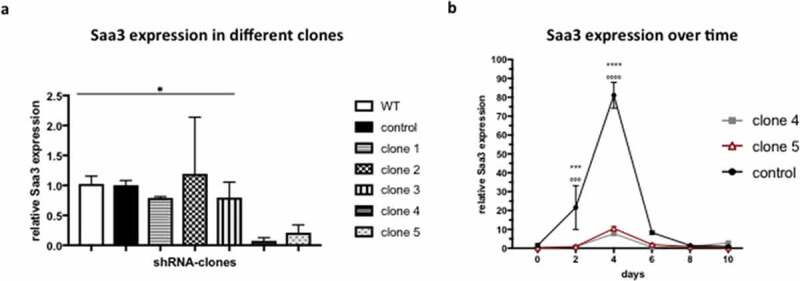
Creation of a Saa3 knockdown cell line. mRNA levels of 3T3-F442A pre-adipocytes that were transfected with lentiviral vectors containing different DNA sequences to silence Saa3 expression. Saa3 expression in clone 4, clone 5, and the control cell line over time (b). Data are presented as mean ± SEM and statistical significance was calculated using a Kruskal-wallis test and a post hoc Dunn’s multiple comparisons test for the comparisons of the different clones, and with a two-way ANOVA for Saa3 expression over time. Results were considered significant when p < 0.05

To investigate the importance of Saa3 in adipogenesis, we first generated a pre-adipocyte cell line with a stable knockdown of Saa3. 5 different clones were compared to the control plasmid SHC002V, which contained no sequence to silence Saa3. One of these five clones produced a significantly significant knockdown ≥ 70% (Figure 1). Therefore, this clone was selected for all further experiments. No significant change in Saa3 expression was observed between the 3T3-F442A pre-adipocytes transfected with the control SHC002V particles and wild-type pre-adipocytes, confirming it as a suitable control (Figure 1).

* Means p < 0.05.

WT, wild type.

### Effect of saa3 gene silencing on in vitro adipogenesis

**Figure 2.**
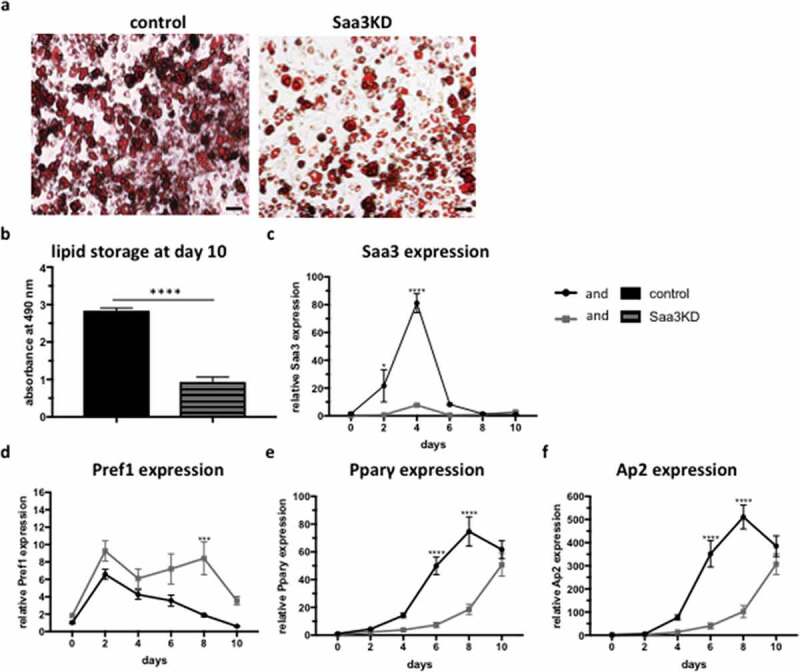
The effect of Saa3 gene silencing on in vitro adipogenesis. Oil-red-O images of control adipocytes (a, left) and Saa3 gene silenced adipocytes (a, right) were taken at 50x magnification and (b) depicts the quantification of the staining. The scale bar corresponds to 100 µm. (c) Saa3, (d) Pref-1, (e) Pparγ and (f) Ap2 mRNA levels of control adipocytes or Saa3 gene silenced adipocytes during the differentiation protocol. Statistical significance was calculated using Mann-whitney U test for the quantification of the Oil red O and two-way ANOVA for expression over time and results were considered significant when p < 0.05. Data are presented as mean ± SEM and n = 3 independent experiments

After establishing a stable Saa3 knockdown pre-adipocyte cell line, the effect of Saa3 gene silencing on adipogenesis was investigated in vitro. Saa3 gene silencing impaired lipid accumulation in differentiated adipocytes (Figure 2a and b). During differentiation, Saa3 knockdown remained stable in the Saa3 gene silenced group, whereas there was a peak in Saa3 expression in the control group at day 4 (Figure 2c). In the Saa3 gene silenced group Pref-1 expression, a marker for pre-adipocytes, was higher than the control group (Figure 2d). Furthermore, there was a lower expression of the adipogenic markers Ap2 and Pparγ in the Saa3 gene silenced group compared to the control group (Figure 2e and f). Taken together, these data demonstrate that gene silencing of Saa3 impairs in vitro adipogenesis.

* Means p < 0.05, ** means p < 0.01, *** means p < 0.001 and **** means p < 0.0001.

KD, knockdown.

**Figure 3.**
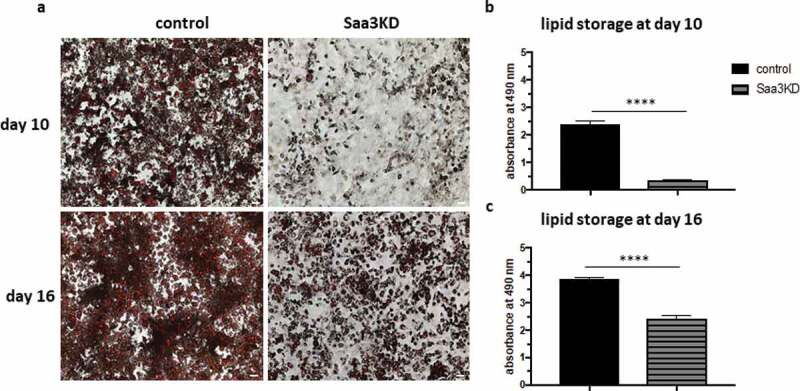
The effect of Saa3 gene silencing on lipid accumulation in a 16-day differentiation protocol. Oil-red-O images of control adipocytes (a, left) and Saa3 gene silenced adipocytes (a, right) were taken on day 10 (top) and on day 16 (bottom) of differentiation at a 50x magnification and (b) depicts the quantification of the staining on day 10, (c) depicts the quantification of the staining on day 16. The scale bar corresponds to 100 µm. Statistical significance was calculated using Mann-whitney U test and results were considered significant when p < 0.05. Data are presented as mean ± SEM and n = 3 independent experiments

At day 10 of the differentiation, we noticed that the expression of the pro-adipogenic markers also increased in the Saa3 gene silenced group, whereas Pref-1 expression decreased (Figure 2d–f). Even though the Oil Red O staining clearly demonstrated less differentiation, we hypothesized that Saa3 gene silencing might not completely impair adipogenesis, but only delays it. Therefore, a second in vitro study was conducted with a longer differentiation of 16 days instead of 10 days. Moreover, since we noticed that Saa3 mRNA levels spiked in the control group at day 4, we decided to add additional time points to get a more detailed perspective on what happens in the early stages of differentiation. In accordance with the previous experiment, we found that at day 10 of the differentiation protocol, there was much less lipid accumulation in the Saa3 gene silenced group than in the control group (Figure 3a and b). At day 16 of the differentiation, there still was less lipid accumulation in the Saa3 gene silenced group compared to the control group, indicating that adipocyte differentiation is indeed impaired and not just delayed by Saa3 gene silencing (Figure 3a and c).

* Means p < 0.05, ** means p < 0.01, *** means p < 0.001 and **** means p < 0.0001.

KD, knockdown.

**Figure 4.**
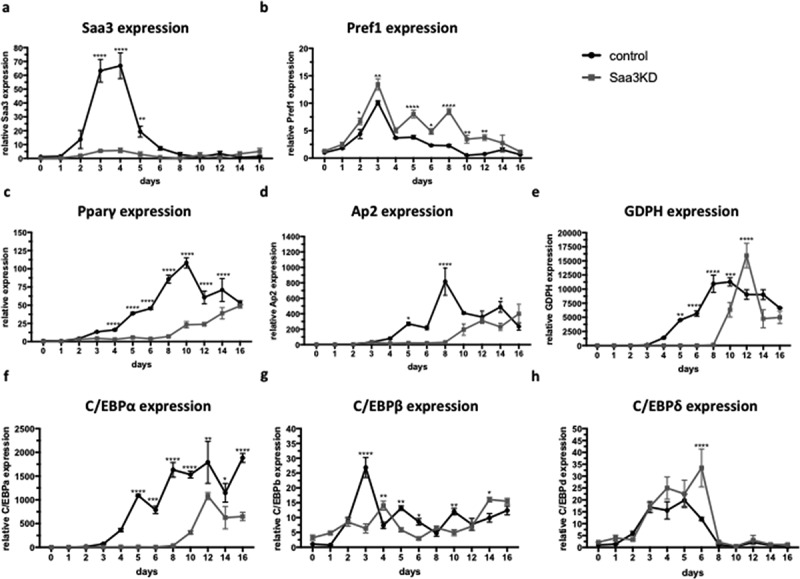
The effect of Saa3 gene silencing on expression levels of adipogenic markers in a 16-day differentiation protocol

In accordance with the previous study, Saa3 expression remained low in the Saa3 gene silenced group and there again was a spike in Saa3 expression from day 3–5 in the control group (Figure 4a). Pref-1 mRNA levels remained higher in the Saa3 gene silenced group than in the control group, indicating impaired adipocyte differentiation (Figure 4b). The spike in Saa3 expression coincides with the spike in Pref-1 expression (Figure 4a and b). Literature demonstrated an inverse correlation between Saa3 and the transcription factor C/EBP. Furthermore, Pref-1 and C/EBP expression are also inversely correlated since Pref-1 is pre-adipocyte marker and C/EBP expression induces differentiation. The relative expression of the pro-adipogenic markers Pparγ, Ap2, GDPH, C/EBPα and C/EBPβ was significantly higher in the control group than in the Saa3 gene silenced group (Figure 4c–g). No clear differences were found between groups in C/EBPδ expression (Figure 4h). Taken together, these data clearly indicate that Saa3 gene silencing impairs adipogenesis in vitro and does not just delay it.

Saa3, (b) Pref-1, (c) Pparγ,(d) Ap2, (e) GPDH, (f) C/EBPα, (g) C/EBPβ and (h) C/EBPδ mRNA levels of control adipocytes or Saa3 gene silenced adipocytes during the differentiation protocol. Statistical significance was calculated using a two-way ANOVA and results were considered significant when p < 0.05. Data are presented as mean ± SEM and n = 3 independent experiments .* Means p < 0.05, ** means p < 0.01, *** means p < 0.001 and **** means p < 0.0001. KD, knockdown.

### Saa3 gene silencing impairs in vivo adipogenesis

Since the results from the in vitro study clearly demonstrate that Saa3 gene silencing impairs adipogenesis in our in vitro adipocyte differentiation model, we next performed a study to confirm these data in our in vivo adipogenesis model. In this model, male, NUDE mice were injected SC in the back with either control pre-adipocytes or Saa3 gene silenced pre-adipocytes. These mice were then put on a western diet for 5 weeks to stimulate fat pad formation.

**Figure 5.**
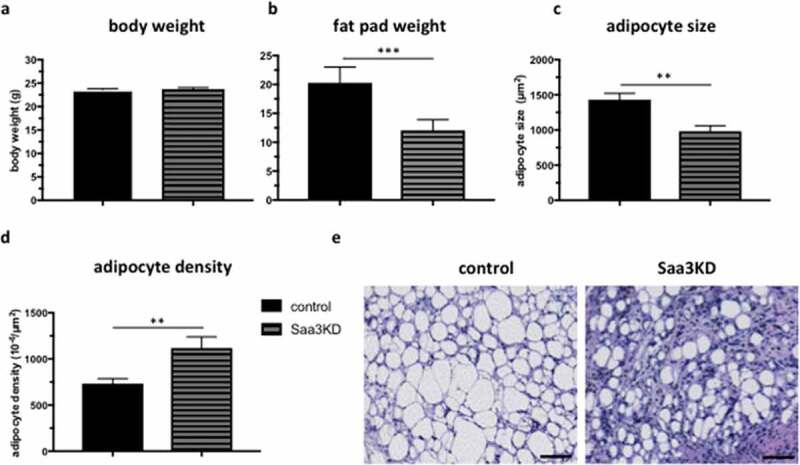
The effect of Saa3 gene silencing on adipocyte size in an in vivo model of adipogenesis

At the end of the dietary period, no difference was found in body weight (Figure 5a). However, weights of the de novo formed fat pads were smaller in the Saa3 gene silenced group than in the control group (Figure 5b). Furthermore, adipocyte size of the de novo formed fat pads was smaller in the Saa3 gene silenced group than in the control group, corresponding to a higher adipocyte density (Figure 5c–e).

Body weight (a), fat pad weight (b), adipocyte size (c), and adipocyte density (d) of *de novo* formed fat pads in NUDE mice that were injected with either control pre-adipocytes or Saa3 gene silenced pre-adipocytes. H&E-stained images (e) of *de novo* formed fat pads were taken at 200x magnification. The scale bar corresponds to 100 µm. Data are represented as mean ± SEM and n = 7 per group. Statistical significance was calculated using Mann-whitney U test and results were considered significant when p < 0.05. * Means p < 0.05, ** means p < 0.01, *** means p < 0.001 and **** means p < 0.0001. KD = knockdown.

**Figure 6.**
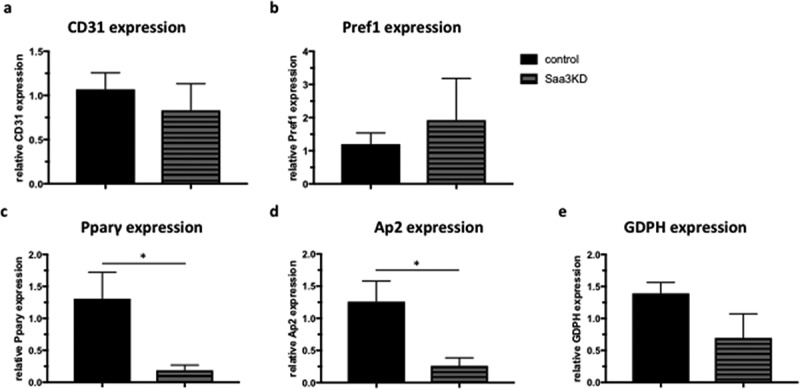
The effect of Saa3 gene silencing on the expression profile of de novo formed fat pads in a murine model of adipogenesis. CD31 (a), Pref1 (b), Pparγ (c), Ap2 (d), and GDDPH (e) mRNA levels in de novo formed fat pads in NUDE mice that were injected with either control pre-adipocytes or Saa3 gene silenced pre-adipocytes. Data are represented as mean ± SEM and n = 8 per group. Statistical significance was calculated using Mann-whitney U test and results were considered significant when p < 0.05

No difference was found in CD31 mRNA levels between groups (Figure 6a). There was a trend towards a higher expression of Pref-1 in the Saa3 gene silenced group compared to the control group (Figure 6b). Additionally, expression of the pro-adipogenic markers Pparγ and Ap2 was lower in the Saa3 gene silenced group than in the control group (Figure 6c and d). Furthermore, there was a trend towards a lower expression of the pro-adipogenic marker GDPH in the Saa3 gene silenced group than in the control group and a trend towards a higher expression of Pref-1 in the Saa3 gene silenced group compared to the control group (Figure 6e). These data confirm that Saa3 gene silencing also impairs in vivo adipogenesis.

* Means p < 0.05, ** means p < 0.01, *** means p < 0.001 and **** means p < 0.0001.

KD, knock down.

## Discussion

Our data clearly demonstrates that Saa3 plays a role in adipogenesis. In contrast to our data, Filippin-Monteira et al. demonstrated that adding recombinant Saa to 3T3-L1 adipocytes inhibited adipocyte differentiation [12]. However, in this study, they never define which isoform of Saa is used and they used a different pre-adipocyte cell line than us, 3T3-L1. We have used the 3T3-F442A cell line in our experiments since this cell line is also used in our in vivo adipogenesis model. Moreover, we have been able to confirm our in vitro data with this in vivo model, indicating that indeed Saa3 is important for efficient adipogenesis, both in vitro and in vivo.

Studies have shown that dexamethasone, a glucocorticoid often used to initiate adipocyte differentiation, induces Saa3 expression [14]. Furthermore, it has been shown that Saa3 induces Pparγ expression [13]. In accordance with these studies, we found much lower Pparγ mRNA levels in the Saa3 gene silenced group than in the control group suggesting that the impaired adipogenesis in the Saa3 gene silenced group is, at least partly, due to a lack of stimulus to induce Pparγ expression.

During the early stages of adipogenesis, Pref-1 induces the expression of a transcription factor, Sox9, which suppresses C/EBPβ and C/EBPδ expression [22]. The addition of dexamethasone and IBMX at day 2 of the differentiation protocol, induces the expression of C/EBPβ and δ, respectively [22–24]. These markers further induce the expression of two key adipogenic factors, C/EBPα and Pparγ [25]. We found that in the control group, there was a peak in C/EBPβ expression at day 3. This was followed by an increase in C/EBPα and Pparγ starting at day 4 and further increasing until day 10 of the differentiation. This peak in C/EBPβ expression was absent in the Saa3 gene silenced group. Subsequently, C/EBPα and Pparγ expression also remained much lower in the Saa3 gene silenced group as compared to the control group throughout the entire experiment. Pref-1 expression remained higher in the Saa3 gene silenced group than in the control group during the entire experiment. Therefore, we postulate that the absence of Saa3 abrogates the drop in Pref-1 expression that normally starts at day 4 in the differentiation protocol, thus inhibiting the differentiation cascade, which is initiated by this Pref-1 decrease. It has been demonstrated that retinoic acid induces pref-1 expression [3,4]. Furthermore, Saa3 has retinol-binding capacity [5]. This gives rise to the hypothesis that in normal adipogenesis, Saa3 is induced, binds retinol and via this route it abrogates Pref-1 expression. This could be the mechanism of the drop in Pref-1. When Saa3 is not present, the retinol is not bound and thus remains active. This results in no drop in Pref-1 and therefore an impairment of adipogenesis. However, more research on this possible mechanism is necessary to confirm this hypothesis. We postulate that the Saa3 gene silenced adipocytes are still able to start expressing pro-adipogenic signals, but in a later stage than wild-type adipocytes. However, since there is no Pref-1 drop in the Saa3 gene silenced adipocytes in the early stages of adipogenesis, we hypothesize that they miss the right signals at the right time point and therefore can no longer differentiate into mature adipocytes efficiently. The fact that they can still start expressing pro-adipogenic markers at a later stage, suggests that there is more than one pathway that can lead to a mature adipocyte, although it is clear from the Oil Red O stainings and the in vivo experiment that these pathways are far less efficient in inducing adipocyte differentiation. This could be a mechanism through which Saa3 interferes with Pparγ expression and thus also impairs adipogenesis, although more research is needed before a definite conclusion can be made regarding this subject. Our results are contradictory to the study by Ather et al, where Saa3 knockout mice had increased adipose tissue and body weights [26]. However, in this study adipocyte size was not determined. The increase in adipose tissue might be, at least in part, be due to an increased lipid storage in the existing adipocytes. Adipocyte hyperplasia has been proposed as a possible rescue mechanism to decrease adipose tissue dysfunction due to adipocyte hypertrophy [1,2]. We hypothesize that the increased metabolic complications in the Saa3 knockout group could thus be caused by excess adipocyte hypertrophy and ectopic lipid depositions because the formation of new adipocytes is impaired. Further research would be necessary to investigate this hypothesis.

The NUDE mice used in the in vivo part of this study are immune deficient since they lack T-cell immunity. There have been some studies highlighting the interaction of Saa3 with T-cell immunity [27,28]. But, the influence of T-cell immunity on adipogenesis seems rather limited. Moreover, consistent with previous studies, our control adipocytes did give rise to the formation of de novo fat pads, confirming that the impaired adipogenesis in the Saa3 gene silenced group is indeed due to a lack of Saa3 [16,20,29].

In conclusion, we demonstrated that Saa3 plays an important role in adipocyte differentiation. This opens new perspectives for Saa3 as a therapeutic target in the battle against obesity.

## Supplementary Material

Supplemental Material

## References

[1] Semenkovich CF. Insulin resistance and atherosclerosis. J Clin Invest. 2006;116(7):1813–1822. 16823479 10.1172/JCI29024 PMC1483180

[2] Ghaben AL, Scherer PE. Adipogenesis and metabolic health. Nat Rev Mol Cell Biol. 2019;20(4):242–258. 30610207 10.1038/s41580-018-0093-z

[3] Manna P, Jain SK. Obesity, oxidative stress, adipose tissue dysfunction, and the associated health risks: causes and therapeutic strategies. Metab Syndr Relat Disord. 2015;13(10):423–444. 26569333 10.1089/met.2015.0095 PMC4808277

[4] Burton DGA, Faragher RGA. Obesity and type-2 diabetes as inducers of premature cellular senescence and ageing. Biogerontology. 2018;19(6):447–459. 30054761 10.1007/s10522-018-9763-7 PMC6223730

[5] Hotamisligil GS. Inflammation and metabolic disorders. Nat Immunol. 2006;444(14):860–867. 10.1038/nature05485 17167474

[6] Osborn O, Olefsky JM. The cellular and signaling networks linking the immune system and metabolism in disease. Nat Med. 2012;18(3):363–374. 22395709 10.1038/nm.2627

[7] Vishvanath L, Gupta RK. Contribution of adipogenesis to healthy adipose tissue expansion in obesity. J Clin Invest. 2019;129(10):4022–4031. 31573549 10.1172/JCI129191 PMC6763245

[8] Longo M, Zatterale F, Naderi J, et al. Adipose tissue dysfunction as determinant of obesity-associated metabolic complications. Int J Mol Sci. 2019;20(9):2358. 10.3390/ijms20092358 PMC6539070 31085992

[9] Buck M, Gouwy M, Wang J, et al. Structure and expression of different Serum Amyloid A (SAA) variants and their concentration-dependent functions during host insults. Curr Med Chem. 2016;23(17):1725–1755. 27087246 10.2174/0929867323666160418114600 PMC5405626

[10] Hartigh LJD, Wang S, Goodspeed L, et al. Deletion of serum amyloid A3 improves high fat high sucrose diet-induced adipose tissue inflammation and hyperlipidemia in female mice. PLoS One. 2014;9(9):e108564. 25251243 10.1371/journal.pone.0108564 PMC4177399

[11] Chait A, Den Hartigh LJ. Adipose tissue distribution, inflammation and its metabolic consequences, including diabetes and cardiovascular disease. Front Cardiovasc Med. 2020;7(February):1–41. 32158768 10.3389/fcvm.2020.00022 PMC7052117

[12] Filippin-Monteiro FB, De Oliveira EM, Sandri S, et al. Serum amyloid A is a growth factor for 3T3-L1 adipocytes, inhibits differentiation and promotes insulin resistance. Int J Obes. 2012;36(8):1032–1039. 10.1038/ijo.2011.193 PMC3419975 21986708

[13] Li H, Zhao Y, Zhou S, et al. Serum amyloid A activates peroxisome proliferator-activated receptor γ through extracellularly regulated kinase 1/2 and COX-2 expression in hepatocytes. Biochemistry. 2010;49(44):9508–9517. 20853849 10.1021/bi100645m

[14] Fasshauer M, Klein J, Kralisch S, et al. Serum amyloid A3 expression is stimulated by dexamethasone and interleukin-6 in 3T3-L1 adipocytes. J Endocrinol. 2004;183(3):561–567. 15590982 10.1677/joe.1.05699

[15] Green H, Kehinde O. Spontaneous heritable changes leading to increased adipose conversion in 3T3 cells. Cell. 1976;7(1):105–113. 949738 10.1016/0092-8674(76)90260-9

[16] Christiaens V, Van Hul M, Lijnen HR, et al. CD36 promotes adipocyte differentiation and adipogenesis. Biochim Biophys Acta. 2012;1820(7):949–956. 22507268 10.1016/j.bbagen.2012.04.001

[17] Ramírez-Zacarías JL, Castro-Muñozledo F, Kuri-Harcuch W. Quantitation of adipose conversion and triglycerides by staining intracytoplasmic lipids with oil red O. Histochemistry. 1992;97(6):493–497. 1385366 10.1007/BF00316069

[18] Mandrup S, Loftus TM, Macdougald OA, et al. Obese gene expression at in vivo levels by fat pads derived from s.c. implanted 3T3-F442A preadipocytes. Proc Natl Acad Sci U S A. 1997;94(9):4300–4305. 9113984 10.1073/pnas.94.9.4300 PMC20717

[19] Neels JG, Thinnes T, Loskutoff DJ. Angiogenesis in an in vivo model of adipose tissue development. Faseb J. 2004;18(9):983–985. 15084517 10.1096/fj.03-1101fje

[20] Scroyen I, Christiaens V, Lijnen H. No functional role of plasminogen activator inhibitor-1 in murine adipogenesis or adipocyte differentiation. J Thromb Haemost. 2007;5(1):139–145. 17067365 10.1111/j.1538-7836.2006.02284.x

[21] Geys L, Roose E, Vanhoorelbeke K, et al. Role of ADAMTS13 in diet-induced liver steatosis. Mol Med Rep. 2017;16(2):1451–1458. 29067443 10.3892/mmr.2017.6714

[22] Hei SS. Minireview: pref-1: role in adipogenesis and mesenchymal cell fate. Mol Endocrinol. 2009;23(11):1717–1725. 19541743 10.1210/me.2009-0160 PMC2775937

[23] Yeh WC, Cao Z, Classon M, et al. Cascade regulation of terminal adipocyte differentiation by three members of the C/EBP family of leucine zipper proteins. Genes Dev. 1995;9(2):168–181. 7531665 10.1101/gad.9.2.168

[24] Farmer SR. Transcriptional control of adipocyte formation. Cell Metab. 2007;4(4):263–273. 10.1016/j.cmet.2006.07.001 PMC1958996 17011499

[25] Zubiría MG, Giordano AP, Gambaro SE, et al. Dexamethasone primes adipocyte precursor cells for differentiation by enhancing adipogenic competency. Life Sci. 2020;261(May):118363. 32861797 10.1016/j.lfs.2020.118363

[26] Ather JL, Poynter ME, Wong GW. Serum amyloid A3 is required for normal weight and immunometabolic function in mice. PLoS One. 2018;13(2):e0192352. 29390039 10.1371/journal.pone.0192352 PMC5794179

[27] Lee JY, Hall JA, Kroehling L, et al. Serum amyloid A proteins induce pathogenic Th17 cells and promote inflammatory disease. Cell. 2020;180(1):79–91.e16. 31866067 10.1016/j.cell.2019.11.026 PMC7039443

[28] Ather JL, Ckless K, Martin R, et al. Serum amyloid A activates the NLRP3 inflammasome and promotes Th17 allergic asthma in mice. J Immunol. 2011;187(1):64–73. 21622869 10.4049/jimmunol.1100500 PMC3119761

[29] Scroyen I, Cosemans L, Lijnen HR. Effect of tissue inhibitor of matrix metalloproteinases-1 on in vitro and in vivo adipocyte differentiation. Thromb Res. 2009;124(5):578–583. 19608218 10.1016/j.thromres.2009.06.020

